# Clinical data needs in the neonatal intensive care unit electronic medical record

**DOI:** 10.1186/1472-6947-14-92

**Published:** 2014-10-24

**Authors:** Marc A Ellsworth, Tara R Lang, Brian W Pickering, Vitaly Herasevich

**Affiliations:** Division of Neonatal Medicine, Mayo Clinic College of Medicine, Rochester, MN USA; Department of Anesthesiology, Mayo Clinic College of Medicine, Rochester, MN USA; Multidisciplinary Epidemiology and Translation Research in Intensive Care (METRIC), Mayo Clinic College of Medicine, Rochester, MN USA

**Keywords:** NICU, Computerized medical record systems, Health information technology, Medical informatics, Electronic health records

## Abstract

**Background:**

The amount of clinical information that providers encounter daily creates an environment for information overload and medical error. To create a more efficient EMR human-computer interface, we aimed to understand clinical information needs among NICU providers.

**Methods:**

A web-based survey to evaluate 98 data items was created and distributed to NICU providers. Participants were asked to rate the importance of each data item in helping them make routine clinical decisions in the NICU.

**Results:**

There were 23 responses (92% – response rate) with participants distributed among four clinical roles. The top 5 items with the highest mean score were *daily weight*, *pH*, *pCO2*, *FiO2,* and *blood culture results.* When compared by clinical role groupings, supervisory physicians gave individual data item ratings at the extremes of the scale when compared to providers more responsible for the daily clinical care of NICU patients.

**Conclusion:**

NICU providers demonstrate a need for large amounts of EMR data to help guide clinical decision making with differences found when comparing by clinical role. When creating an EMR interface in the NICU there may be a need to offer options for varying degrees of viewable data densities depending on clinical role.

## Background

The combination of continuous monitoring and the ability of the electronic medical record (EMR) to store large amounts of data creates a potential for information overload in the intensive care (ICU) setting [1]. The potential dangers underlying this information overload relate to the inability of practitioners to discern pertinent from irrelevant information [2] and the accumulation of errors of cognition and performance associated with data corruption [3, 4].

A possible way to combat the risks of information overload may center on the development and implementation of advanced health information technologies (HITs). The Institute of Medicine and the United States Department of Health and Human Services have both advocated for the enhanced creation and use of efficient EMRs [5]. Recently, the HIT for Economic and Clinical Health (HITECH) Act was enacted which has allocated federal funds to aid in this endeavor [6].

Previous EMR implementation experiences in academic institutions have been met with conflicting outcomes. Although most institutions have demonstrated improved outcomes, increased productivity, and fewer errors [3, 7–10] associated with EMR use, there is still guarded optimism on how best to design and integrate EMRs into clinical practice [11].

The development of a novel EMR human-computer interface, Ambient Warning and Response Evaluation (AWARE), at our institution resulted in improved performance and decreased errors of cognition in the adult ICU setting when compared to the standard EMR system [3]. The creation of this specific interface was based on expert panel input and data utilization models designed to assess the specific information needs of the unit [12, 13]. This design methodology is in contrast to the vendor-generated platforms most commonly used in hospital EMRs [14].

The critically ill pediatric population is a unique group with specific medical information needs [15, 16]. Additionally, there are special considerations that need to be taken into account when creating EMR interfaces for use specifically in the neonatal intensive care unit (NICU) [17]. As a result there have been standards suggested to help guide the creation of pediatric and neonatal specific EMRs [18, 19]. The creation of these guidelines underscore the importance of creating efficient EMR human-computer interfaces, such as AWARE, to help decrease medical errors and improve clinical care.

In order to create an end-user designed patient-centered EMR interface we endeavored to better understand the exact clinical information needs of NICU practitioners. In the present study we address this gap in knowledge by a survey to determine what data NICU providers find useful in clinical decision making for inclusion into future EMR human-computer interfaces.

## Methods

### Study design

A web-based survey was conducted at Mayo Clinic, Rochester, MN, an academic tertiary health care center, equipped with a comprehensive EMR. The Mayo Clinic NICU has 26 level III beds and admits approximately 350 infants per year. The survey was conducted among NICU providers of varying clinical roles. The study was approved by the Institutional Review Board (IRB) at Mayo Clinic. The study was deemed exempt from consent requirements by the IRB (13–003930).

### Study subjects

Twenty-five subjects for the survey were selected from 4 clinical role designations; attending physicians (AP), neonatal fellows (NF), neonatal nurse practitioners (NP), and pediatric residents (PR). The AP group consisted of staff neonatologists and 1 neonatal hospitalist. NPs with significant clinical duty commitments (>50% of shifts occurring in the level III setting) were invited to participate. The PR group consisted of senior pediatric residents with NICU experience within the last year.

### Data collection

#### Instruments

An expert panel, consisting of 2 APs and 1 NF, reviewed our current EMR and identified 98 unique data items that are available to clinical users. Using a 7-point Likert scale [ranging from *not needed* (0) to *absolutely necessary* (6)] subjects were asked to rate each of the 98 data items according to their opinion as to its importance in helping make routine clinical decisions in the NICU. Of note, only a 0 (*not needed*) or 6 (*absolutely necessary*) score was given a categorical description. Scores between these extremes (1–5) were exclusively numerical in nature.

#### Procedures

The survey utilized the Research Electronic Data Capture (REDCap) web-based application [20] and was distributed to the study participants via an embedded e-mail link to the survey. Two e-mail reminders were generated to enhance survey participation. The identity of participants and survey results were kept confidential from all subjects and investigators.

### Data analysis

Survey responses were collected and tabulated by the REDCap tool. The mean score (MS) was calculated for each data item with items then ranked in descending order of MS for generation of the median value and interquartile ranges. The percentage of participants ranking each individual data item as *not needed* or *absolutely necessary* was also determined. The MS for each data item was also stratified according to the respondent’s clinical role. For these analyses APs and NFs were grouped together (AP/NF) as they mainly perform supervisory roles, with NPs and PRs being grouped (NP/PR) as they often are charged with carrying out most pre-rounding, rounding, and post-rounding duties.

### Statistics

All descriptive statistics and comparison of means (Wilcoxon’s rank sum test) were performed in JMP (*v* 9.0.1, SAS Institute, Cary, NC); *p* < .05 was considered statistically significant. TABLEAU^®^ software (Seattle, WA) was used for data visualization. Study format, design, and statistical analyses were done in accordance with similar published studies performed at our institution and under the guidance of statistical support [12, 21].

## Results

Twenty-five survey requests were distributed with 23 responses obtained, giving a response rate of 92%. All 8 APs and 2 NFs completed the survey with 4 NPs and 9 PRs participating in the study.

**Figure 1:**
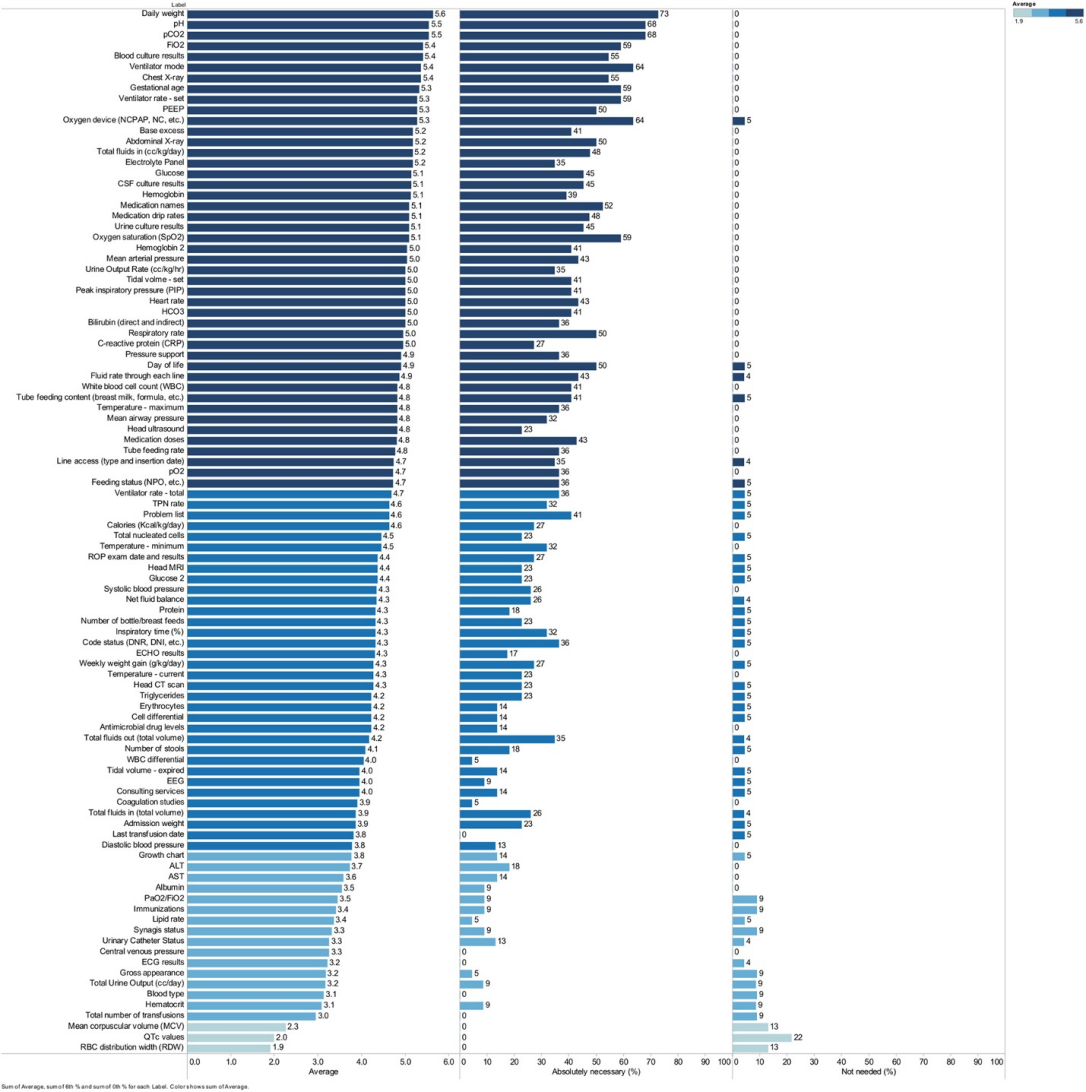
**Data item ratings.** Data items in descending order of the mean score (average) as rated by NICU providers to the perceived importance in guiding clinical care. Proportional quartiles are demonstrated by shading of the graph bars. Also shown are the percentages of respondents that rated each data item as *absolutely necessary* and *not needed.*

Figure 1 shows each of the 98 data items listed by descending MS. The top 5 data items with the highest MS were *daily weight*, *pH*, *pCO2*, *FiO2,* and *blood culture results*. The median MS was 4.5 (maximum 6) with 71% of the data items falling within the top 2 quartiles when distributed by proportional quarters. The lowest 5 rated data items were *RBC distribution width (RDW), QTc value*, *mean corpuscular volume (MCV), total number of transfusions,* and *hematocrit*.

Also displayed in Figure 1 is the percentage of respondents that rated each item as *absolutely necessary* or *not needed. Daily weight* received the highest percentage of responders (73%) rating it as an *absolutely necessary* data item. Sixteen of the 98 items (16%) received an *absolutely necessary* rating by more than 50% of respondents. The 3 items with the lowest MS also received the highest percentage of respondents rating each data item as *not needed.* Only 1 item (*QTc value*) was rated by more than 20% of respondents as *not needed.*

Stratifying individual data item MSs by clinical role groupings (AP/NF versus NP/PR) resulted in an alteration of the order of highest ranking data items (Table 1). Seven data items (*daily weight, pH, pCO2, FiO2, blood culture results, ventilator mode, and chest X-ray)* were among the top 10 rated items in both groupings. Similarly, 6 data items (*total urine output, hematocrit, blood type, MCV, RDW, and QTc value*) were among the 10 lowest rated items in both groups.

**Table 1:** **Top ten data items by clinical role**

AP/NF		NP/PR	
Data item	MS	Data item	MS
Daily weight	5.7	Daily weight	5.6
*Oxygen device (i.e. NCPAP)*	*5.7*	pH	5.5
pCO2	5.7	pCO2	5.4
FiO2	5.7	FiO2	5.2
Blood culture results	5.7	Blood culture results	5.2
*Ventilator rate - set*	*5.7*	*Gestational age*	*5.2*
Ventilator mode	5.7	Ventilator mode	5.1
Chest X-ray	5.7	Chest X-ray	5.1
*Medication names*	*5.7*	*Total fluids in (cc/kg/day)*	*5.1*
pH	5.6	*PEEP*	*5.0*

A listing of the top 10 data items with the largest mean score (MS) stratified by clinical role groupings. Data items that differ between the groups are *italicized*.

**Figure 2:**
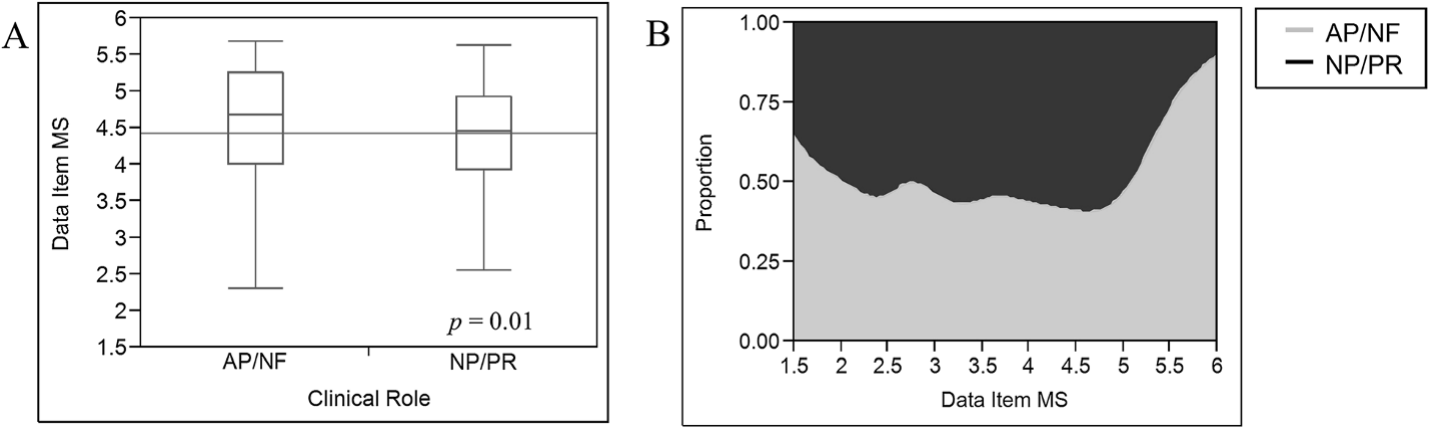
**Variance by clinical role. (A)** Variance in the individual data item mean scores stratified by clinical role (AP – attending physician; NF – neonatal fellow; NP – nurse practitioner; PR – pediatric resident). Data are displayed in box-whisker graphs (median, interquartile range, total range) with the lighter horizontal line showing the grand mean. **(B)** Proportion of density chart illustrating the distribution of data item mean scores by clinical role (no statistical analyses performed).

Figure 2 shows the results of the distribution of MSs for every data item stratified by clinical role groupings. The AP/NF group rated data items significantly higher than the NP/PR group with the overall means of all data item MSs being 4.5 and 4.3 respectively (*p = 0.01)*. Figure 2 also illustrates the same data analyses displayed in a proportion of densities chart (no statistical analyses performed). This chart demonstrates that the AP/NF group generated more data item MSs at the extremes of the scale with a larger proportion of both higher *and* lower rated data items compared to NP/PRs, who produced a larger proportion of moderately rated data items.

## Discussion

We conducted a survey among NICU providers to assess the clinical information needs in an effort to create a more effective EMR human-computer interface. To our knowledge this is the first study attempting to assess these specific needs in a systematic way. Our survey had a high response rate with participants distributed among 4 distinct clinical role designations. The data demonstrates that when making clinical decisions NICU providers rely on a significant proportion of the large amounts of objective data provided to them by the EMR. This is evidenced by the fact that nearly three-fourths of the data items were ranked within the top 2 proportional quartiles with only 3 items contained in the bottom quartile. In addition, there was a large difference at the extremes of the rankings with many more respondents ranking items as *absolutely necessary* as compared to *not needed*. This finding of high EMR-derived data needs is consistent with the finding that NICU providers prefer objective data compared to verbal communication or clinical notes as their primary clinical information source [22].

The distribution of scores aligns with what one would expect clinically. Of the 10 highest rated data items, 7 were related to a respiratory parameter, one often associated with ventilatory management. It is well known that a significant number of major morbidities among NICU patients are respiratory in nature with a significant amount of daily management related to this system [23, 24]. As well, the highest rated item, *daily weight*, is vital for daily medication and fluid calculations and is marked as one the most fundamental data items required in the development of pediatric HITs [18, 19].

In addition to our findings when respondents were grouped as a whole, there were important findings when data item ratings were stratified by clinical role. By grouping in this manner (AP/NF and NP/PR) we attempted to better understand the clinical information needs of users with similar clinical responsibilities. The results of these comparisons have clinical practicality and can offer insights into the appropriate development of HITs best suited to address differing clinical needs. NP/PRs are often responsible for most pre-rounding and rounding duties and are the primary care provider of NICU patients throughout a day [25–27]. As a result, providers in this role have a need for access to large amounts of data items, without much discrimination of importance, in order to collect all the necessary information for dissemination in multidisciplinary patient rounds and in the clinical note. In contrast, supervisory physicians (AP/NFs) often give a disproportionate significance to a smaller set of data items that aid them in making the most critical decisions and care plans with certain data items being lightly regarded or even ignored.

These descriptions of differing roles are supported by our study as AP/NF data item ratings generated bimodal peaks at the extremes of the scale with a more consistent and evenly distributed rating pattern produced by NP/PRs. These findings may suggest that appropriate EMR interfaces for the NICU setting should include different viewing options that are catered to the primary clinical role of the user. Interfaces for supervisory physicians may include less data items that highlight selected, critically relevant data while those for NPs and PRs may be similar to current EMR interfaces with most patient data items readily available and viewable. Further studies are needed to verify these findings and better elucidate what specific data items should be included in a proposed limited data option that can be utilized by supervisory physicians and others (i.e. consulting services), where large amounts of data can be safely omitted without patient care compromise.

Our study has some limitations which include the relatively small number of participants and a single institution survey. Our survey included 23 participants mainly due to the relatively low volume and staffing needs of our NICU. However, we were able to include every AP and NF with a large proportion of NP and PR participation. Despite the low absolute numbers, we feel these survey results accurately reflect the opinions of the care providers and can be used to help guide interface development. In addition, it is important to recognize that these results may reflect a local practice and should be generalized to other settings with these limitations in mind. Regardless, in the development of patient-centered clinical tools, the clinician’s perspective from a single hospital play a more important role than population based observations.

Another limitation of this study involves asking respondents to rate the importance of data items in helping make “routine clinical decision”. We are aware that there are often times when clinical needs reach beyond the scope of what would be considered “routine” and require a different data requirement. It would be difficult to speculate the change in ratings, if any, if the word “routine” were removed from the survey. Regardless, we wanted the results of the survey to portray the ideas of providers in the most common and frequent clinical scenarios and designed the survey as such. We feel that options for viewing different levels of data density, as discussed earlier, would be a way to overcome this obstacle and provide adequate levels of data catered to each clinical situation.

This is the first step in a process of creating and adapting an EMR human-computer interface that involves a design allowing for end-users to influence its final product. A methodical process such as this is important in creating an environment where the implementation of new technology is well accepted and tailored to the needs of the users [28]. In addition, the design of our study creates an opportunity for replication in other centers in an effort to compare and contrast the information needs among providers in various NICU settings.

## Conclusion

Our study illustrates the myriad of data items available to NICU caregivers for use in clinical decision making and demonstrates that providers at our institution feel that a majority of those data items are important with significant differences found when comparing by clinical role. This creates the need to develop patient-centered EMR human-computer interfaces and other HITs that present vast amounts of data [1] in a way that is easily synthesized [17] and offers options for varying degrees of viewable data densities depending on clinical role. In this way one can better create EMR interfaces that are relevant to the clinical expectations of providers while at the same time achieve a goal of reducing information overload and lowering the risk for medical errors [3, 13].

## Abbreviations

AP: Attending physician
AWARE: Ambient warning and response evaluation
EMR: Electronic medical record
HIT: Health information technology
MS: Mean score
NICU: Neonatal intensive care unit
NF: Neonatal fellow
NP: Neonatal nurse practitioner
PR: Pediatric resident.
